# Interleukin-17A pretreatment attenuates the anti-hepatitis B virus efficacy of interferon-alpha by reducing activation of the interferon-stimulated gene factor 3 transcriptional complex in hepatitis B virus-expressing HepG2 cells

**DOI:** 10.1186/s12985-022-01753-x

**Published:** 2022-02-10

**Authors:** Jiaxuan Zhang, Kai Liu, Gaoli Zhang, Ning Ling, Min Chen

**Affiliations:** 1 Department of Infectious Diseases, Institute for Viral Hepatitis, Key Laboratory of Molecular Biology for Infectious Diseases (Ministry of Education), Second Affiliated Hospital of Chongqing Medical University, Chongqing, China; 2 Department of Clinical Laboratory, The People’s Hospital of Leshan, Chongqing, China

**Keywords:** Hepatitis B virus, Interferon alpha, Interleukin-17A, Type I interferon signaling pathway

## Abstract

**Background:**

Some cytokine signaling pathways can interact with interferon (IFN)-α pathway and thus regulate cell responses to IFN-α. Levels of the pro-inflammatory cytokine interleukin-17A (IL-17A) were found to be elevated in both the peripheral blood and liver in chronic hepatitis B (CHB) patients. However, how IL-17A affects the anti-HBV activity of IFN-α remains unclear.

**Methods:**

The effects of IL-17A on anti-HBV activity of IFN-α were evaluated in HBV-expressing HepG2 cells (HepG2-HBV1.3) with IL-17A pretreatment and IFN-α stimulation. Culture supernatant levels of HBsAg, HBeAg, and HBV DNA, or intracellular expression of HBsAg and HBcAg were detected by ELISA, real-time quantitative PCR (RT-qPCR), or western blotting (WB). The expression of canonical IFN-α signaling pathway components, including the interferon-α/β receptor (IFNAR), Janus Kinase 1 (JAK1), Tyrosine Kinase 2 (TYK2), the Interferon Stimulated Gene Factor 3 complex (ISGF3) and IFN-stimulated genes (ISGs), was also examined by RT-qPCR, Immunofluorescence or WB. The effects of IL-17A were further investigated by the suppression of the IL-17A pathway with a TRAF6 inhibitor.

**Results:**

Compared to IFN-α stimulation alone, IL-17A pretreatment followed by IFN-α stimulation increased the levels of HBsAg, HBeAg, and HBV DNA, and decreased the levels of ISGF3 complex (phosphorylated (p)-signal transducer and activator of transcription (STAT1)/p-STAT2/IRF9) and antiviral-related ISGs (ISG15, ISG20 and Mx1). Interestingly, IL-17A pretreatment increased the expression of suppressor of cytokine signaling (SOCS) 1, SOCS3 and USP18, which were also the ISGs negatively regulating activity of ISGF3. Moreover, IFNAR1 protein expression declined more sharply in the group with IL-17A pretreatment than in the group with IFN-α stimulation alone. Blocking the IL-17A pathway reversed the effects of IL-17A on the IFN-α-induced activation of ISGF3 and anti-HBV efficacy.

**Conclusions:**

Our results demonstrate that IL-17A pretreatment could attenuate IFN-α-induced anti-HBV activity by upregulating negative regulators of the critical transcriptional ISGF3 complex. Thus, this might be a potential target for improving response to IFN-α therapy.

**Supplementary Information:**

The online version contains supplementary material available at 10.1186/s12985-022-01753-x.

## Introduction

Interferon-alpha (IFN-α) has multiple biological functions, including direct anti-viral effects, modulation of innate and adaptive immune responses, and suppression of cell proliferation. Clinically approved to treat chronic hepatitis B (CHB) for approximately 30 years, IFN-α exerts therapeutic effects through its direct anti-HBV activity in hepatocytes and immunomodulatory activity on immune cells [1]. Compared with nucleos(t)ide analogs, IFN-α has the advantages of finite treatment duration and high HBeAg- and HBsAg-clearance rates (35% and 3%, respectively). However, its treatment efficiency varies a lot among CHB patients due to both viral and host factors. Although several baseline or on-treatment parameters were demonstrated to be predictors of the response to IFN-α therapy, the mechanisms underlying an inadequate response to IFN-α treatment have not yet been fully clarified [2, 3].

IFN-α can bind the IFN-α/β receptor (IFNAR) at the cell surface and activate the intracellular Janus kinase (JAK)-signal transducers and activators of transcription (STAT) signaling pathway, leading to the tyrosine phosphorylation of STAT1 and STAT2. Finally, a transcription factor complex termed IFN-stimulated gene factor 3 (ISGF3), consisting of a p-STAT1: p-STAT2 dimer and IFN regulatory factor 9 (IRF9), is assembled. Then, ISGF3 enters the nucleus and binds the IFN-stimulated response element (ISRE) to activate the transcription of a wide variety of IFN-stimulated genes (ISGs) [4]. The cell responses to IFN-α are regulated by regulatory ISGs and other host, pathogenic and environmental factors. Among these regulatory factors, inflammatory cytokines and chemokines have been demonstrated to augment or suppress type I IFN signaling. For example, Tian et al. reported that IL-1β attenuates IFN-α/β signaling by directly decreasing STAT1 binding and tyrosine phosphorylation through a proteasome-dependent mechanism [5]. Additionally, TNF-α and IL-10 attenuate IFN-α signaling by reducing the tyrosine phosphorylation of STAT1 through stimulating the expression of negative regulatory proteins, such as SOCS2, SOCS3, and SHP2 [6, 7].

Chronic liver inflammation in CHB results in significant elevation in several inflammatory cytokines, such as IFN-γ, TNF-α, IL-1β, IL-6, IL-17A, and IL-23. Interleukin-17A (IL-17A, or IL-17)), a member of the IL-17 family (IL-17 A ~ F), is a pro-inflammatory cytokine produced mainly by Th17 cells [8]. In CHB patients, the levels of IL-17A in the serum and liver are dramatically increased [9–12]. Serum level of IL-17A was significantly decreased after PEG-IFN-α or nucleoside analog treatment (lamivudine and entecavir) [12–15]. More importantly, the decrease in serum IL-17A levels was more pronounced in the HBeAg-clearance group or the group with a rapid decline in HBsAg (> 1 log10 IU/ml) than in the persistently HBeAg-positive group or the group with a slow decrease in HBsAg (< 1 log10 IU/ml) respectively [12]. Some previous studies showed that IL-17A could both upregulate phosphorylation of the STAT1 protein in lung adenocarcinoma [16] and significantly decrease the mRNA expression of IRF9 in mouse primary hepatocytes [17]. These studies indicate possible regulation of the IFN-α signaling pathway by IL-17A.

In this study, we aimed to observe the effect of IL-17A on the anti-HBV efficacy of IFN-α, with a particular focus on its direct antiviral effect, in HBV-expressing HepG2 cells in vitro. We also further explored the possible mechanism of this regulatory effect. This study would provide new lines of investigation regarding the relationship between inflammatory cytokines and IFN-α treatment efficacy in CHB patients.

## Materials and methods

### Cell line, plasmid, and transfection

HepG2 cells were obtained from the Cell Bank of the Chinese Academy of Sciences (Shanghai, China). They were cultured in high-glucose DMEM (Gibco, USA) supplemented with 10% heat-inactivated fetal calf serum (Gibco) and antibiotics in a humid environment with 5% CO_2_ at 37 °C. The pcDNA-HBV1.3 plasmid, containing a 1.3-unit length HBV genome (ayw subtype) in the pcDNA3.1 backbone, was constructed and preserved at our institute. Briefly, a 4.1 kb fragment of 1.3-fold-overlength HBV genome, derived from pGEM-11zf-HBV1.3 (kindly provided by Dr Protzer, University of Heidelberg, Germany), was cloned into the eukaryotic expression vector pcDNA3.1 at the HindIII site to construct the recombinant plasmid pcDNA3.1-HBV1.3. In pcDNA3.1-HBV1.3 plasmids, CMV promoter of pcDNA3.1 vector only drives synthesis of the pregenomic RNA (pgRNA), whereas subgenomic RNAs are produced from HBV genome endogenous promoters. pcDNA3.1-HBV1.3 plasmid has been verified to be effectively expressed and broadly used in cell lines and mouse models both in our institute and other labs [18–20]. HepG2 cells were suspended at a final concentration of 5.5 × 10^5^ cells/well in 6-well plates and transfected with 4 μg of pcDNA-HBV1.3 plasmid using Lipofectamine 3000 transfection reagent (Invitrogen, USA) according to the protocol supplied by the manufacturer. In our experiments, HepG2-HBV1.3 cells were collected at 48 h after transfection for subsequent experiments. And the expression of HBsAg and HBeAg in the culture supernatant was sustained till 120 h post-transfection (Additional file 3: Fig. S1).

### Treatment with IL-17A and IFN-α

2.5 × 10^5^ HepG2-HBV1.3 cells per well were plated in 12-well plates and cultured for 24 h until they reached approximately 50% confluency. They were first pretreated with IL-17A (PeproTech, UK) at different concentrations for different durations and then cultured with recombinant human IFN-α-2b (1000 IU/ml) (Anhui Anke Biotechnology, China) and IL-17A for additional 24 or 48 h. The cell culture supernatant was collected to assay HBsAg, HBeAg, and HBV DNA levels. At the same time, total RNA and protein were extracted from these cells to detect intracellular HBV markers, IFN-α signal transduction molecules, and ISGs.

### Treatment with the TRAF6 inhibitor C25-140

The cells were plated as previously described. They were first incubated with C25-140 (5 or 20 µM) (MedChemExpress LLC, USA) for 6 h and then treated with IL-17A (50 ng/ml) (PeproTech, UK) for 24 h, followed by combined treatment with recombinant human IFN-α-2b (1000 IU/ml) (Anhui Anke Biotechnology, China) and IL-17A for another 24 h. The cell culture supernatant was collected to assay HBsAg and HBeAg levels. At the same time, total RNA and protein were extracted from these cells to detect intracellular HBV markers, IFN-α signal transduction molecules, and IFN-α-stimulated genes.

### Assays to detect HBsAg, HBeAg, and HBV DNA in the cell culture supernatant

According to the manufacturer’s instructions, levels of HBsAg and HBeAg were detected with commercial ELISA kits (Kehua, China). HBV DNA was extracted, purified with a commercial detection kit (DAAN Gene Co., Ltd., China), and detected by RT-qPCR using the LightCycler™ system (Roche Diagnostics, Switzerland). The levels of them are expressed as a percentage relative to those in mock-treated cells.

### Real-time quantitative PCR (RT-qPCR) analysis of mRNA expression

Total RNA was prepared by the TRIzol extraction method (Invitrogen, USA). Then, RNA (~ 1 μg) was reverse transcribed with an oligo(dT) primer using the PrimeScript RT Reagent Kit with gDNA Eraser (TaKaRa Bio, Japan), and RT-qPCR was performed using SYBR Premix Ex Taq II kit (TaKaRa Bio). The sequences of primers specific for GAPDH (the reference) and target genes are listed in Additional file 1: Table S1. Fold changes in the mRNA expression of target genes were normalized to GAPDH mRNA expression by the 2^−ΔΔCt^ method. The results are presented as the fold induction relative to mock-treated cells.

### Western blotting (WB)

According to the manufacturer’s instructions, whole-cell protein extraction was obtained by using passive cell lysis (Promega, USA) with protease and phosphorylated protease inhibitors. The protein concentration was determined by the BCA method. Samples containing approximately 30 μg of protein per well were separated using SDS-PAGE (12% gel), and then the proteins were transferred to a PVDF membrane (Millipore, USA). The membranes were blocked for 2 h in 5% w/v low-fat dry milk at room temperature in PBST and incubated with primary antibodies overnight at 4 °C. Then, the blots were incubated with HRP-conjugated secondary antibody for 2 h at room temperature and visualized using ECL Plus reagent (Millipore, USA). β-Actin (ACTB) was used as the loading control. Band intensities were semi quantified with ImageJ software, and the gray values were normalized to the value for ACTB. Relative protein expression was defined as the fold change in expression compared to expression in mock-treated cells.

The following antibodies were used: rabbit anti-STAT1 (D1K9Y), rabbit anti-phosphorylated (p-)STAT1-Tyr701 (58D6), rabbit anti-STAT2 (D9J7L), rabbit anti-p-STAT2-Tyr690 (D3P2P), rabbit anti-IRF-9 (D2T8M), rabbit anti-p-JAK1- Tyr1034/1035 (3331S), rabbit anti-p-Tyk2-Tyr1054/1055 (D7T8A) and rabbit anti-ACTB (13E5) from Cell Signaling Technology (USA) and horse anti-HBsAg-ad/ay (ab9193), mouse anti-HBcAg (14E11), rabbit anti-IFN-α/β receptor 1 (IFNAR) (EPR6244), rabbit anti-horse IgG H + L-HRP, goat anti-rabbit IgG H + L-HRP, and goat anti-goat IgG H + L-HRP from Abcam (USA).

### Immunofluorescence staining

Cells were fixed with 4% formaldehyde for 20 min and then treated with cold 100% methanol at − 20 °C for 10 min. Specimens were blocked in 5% BSA (in PBST buffer) for 90 min. Then, the cells were incubated with diluted primary antibody at 4 °C overnight. After thorough washing in PBST, the cells were incubated in diluted fluorochrome-conjugated secondary antibody for 2 h at room temperature in the dark. After incubation with DAPI (1 μg/ml), the cells were observed under a confocal microscope (Nikon, Japan). The following primary and secondary antibodies purchased from Cell Signaling Technology (USA) were used: rabbit anti-p-STAT1-Tyr701 (58D6), rabbit anti-p-STAT2-Tyr690 (D3P2P), rabbit anti-IRF-9 (D2T8M), and anti-rabbit IgG (Alexa Fluor 488 conjugate).

### Statistical analyses

The Prism statistical software package (version 7.0; GraphPad Software Inc., USA) and Statistical Package for Social Sciences version 18.0 (SPSS PASW Statistics version 18.0; SPSS Inc., USA) were used for statistical analyses. For data with normal distribution, the differences were assessed with unpaired t-test (two groups), or one-way ANOVA and Tukey’s test (multiple groups). For data with non-normal distribution, group comparisons were performed by Mann–Whitney U test (two groups) or kruskal–wallis H test (multiple groups). A two-sided P < 0.05 was used to indicate statistical significance. All experiments were performed at least three times.

## Results

### Dose- and time-dependent effects of IL-17A pretreatment on the anti-HBV activity of IFN-α

First, we analyzed a GEO dataset (GEO89610), containing data from human liver hepatocellular carcinoma cells treated with IL-17A. The results of gene set enrichment analysis strongly suggested that IL-17A negatively regulated the IFN-α pathway (Additional file 4: Fig. S2, Additional file 2: Table S2).

**Fig. 1:**
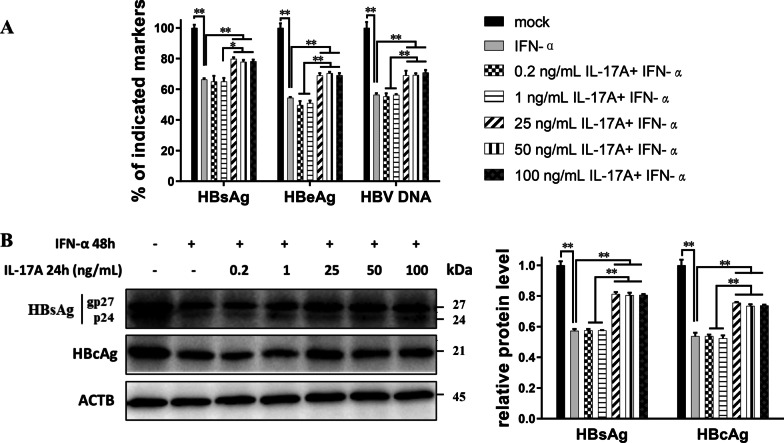
Effect of different concentration of IL-17A on the anti-HBV activity of IFN-α. HepG2-HBV1.3 cells were pretreated with or without altered concentrations of IL-17A (0, 0.2, 1, 25, 50 or 100 ng/ml) for 24 h, followed by co-treatment with or without 1000 IU/ml IFN-α for 48 h. **A** Culture supernatants were collected for assay of levels of HBsAg, HBeAg or HBV DNA. Data were displayed as a percentage of the values obtained for mock-treated cells. **B** Cell lysates were prepared for HBs and HBc protein by western blotting. Relative expression of protein was shown as fold-change in comparison with the mock-treated cells. All data were shown as mean ± standard deviation (SD) (error bars) from at least 3 independent experiments. p < 0.05 is considered statistically significant. *p < 0.05, **p < 0.01 between two indicated groups

Then, the regulatory effect of IL-17A was observed in HepG2-HBV1.3 cells, a cell model which could support HBV expression and replication [18]. Concentrations of IL-17A were set as low-dose (0.2 or 1 ng/ml) and high-dose (25, 50, or 100 ng/ml). IL-17A or IFN-α treatment alone did not significantly affect the cell viability of HepG2-HBV1.3 cells (Additional file 5: Fig. S3 A, B). And the supernatant levels of HBsAg, HBeAg, or HBV DNA were not significantly changed by IL-17A alone at different concentrations for different durations (Additional file 5: Fig. S3 C, D). As shown in Fig. 1, IFN-α (1000 IU/ml) treatment for 48 h efficiently decreased the supernatant levels of HBsAg, HBeAg, and HBV DNA. Compared to IFN-α treatment, pretreatment with IL-17A at relatively high concentrations (25, 50, or 100 ng/ml) significantly elevated the levels of HBV markers and HBV DNA in both the supernatant and cell pellet, but these results were not observed in groups treated with low-dose IL-17A (0.2 or 1 ng/ml). However, no significant difference in HBV markers or HBV DNA expression was found among the 25, 50, and 100 ng/ml IL-17A groups (Fig. 1).

**Fig. 2:**
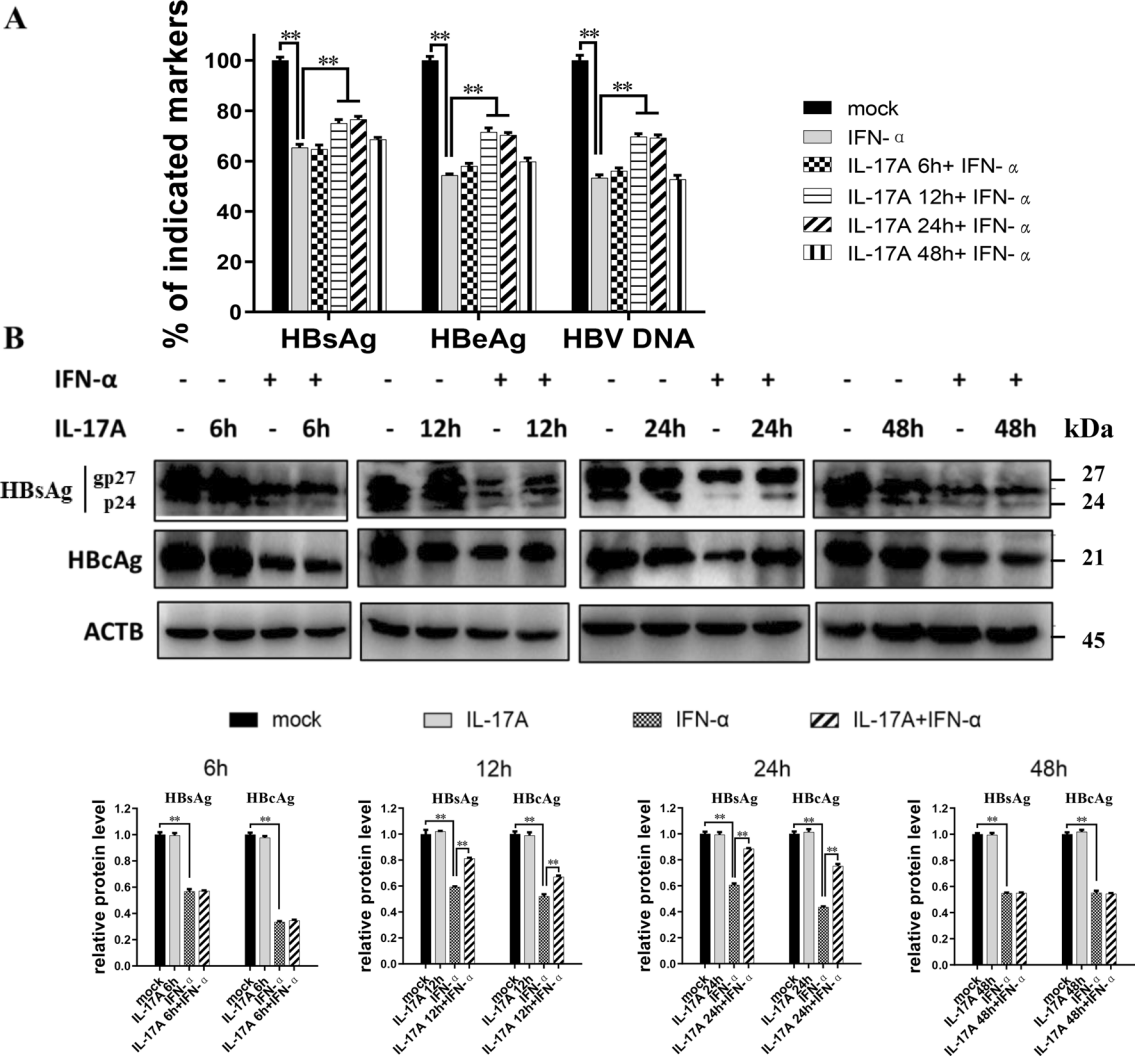
Time-dependent effect of IL-17A pretreatment on the anti-HBV activity of IFN-α. HepG2-HBV1.3 cells were pretreated with or without 50 ng/ml IL-17A for indicated time (6, 12, 24, or 48 h), followed by co-treatment with or without 1000 IU/ml IFN-α for 48 h. **A** Culture supernatants were collected for assay of levels of HBsAg, HBeAg or HBV DNA. Data were displayed as a percentage of the values obtained for mock-treated cells. **B** Cell lysates were prepared for HBs and HBc protein by western blotting. Relative expression of protein was shown as fold-change in comparison with the mock-treated cells. All data were shown as mean ± standard deviation (SD) (error bars) from at least 3 independent experiments. p < 0.05 is considered statistically significant. *p < 0.05, **p < 0.01 between two indicated groups

Then, we evaluated the influence of different durations of 50 ng/ml IL-17A pretreatment on the anti-HBV activity of IFN-α in HepG2-HBV1.3 cells. In the 6 h pretreatment group, no significant difference in the level of supernatant HBV markers or protein expression of HBs and HBc was observed when compared to those in the IFN-α group. When the pretreatment time increased, the 12 h or 24 h group displayed significantly higher levels of these parameters than the IFN-α-treated group. However, when the IL-17A pretreatment time was extended to 48 h, no significant difference in any parameters was found (Fig. 2).

### IL-17A pretreatment reduced mRNA and protein expression of the IFN-α receptor 1 after IFN-α stimulation

**Fig. 3:**
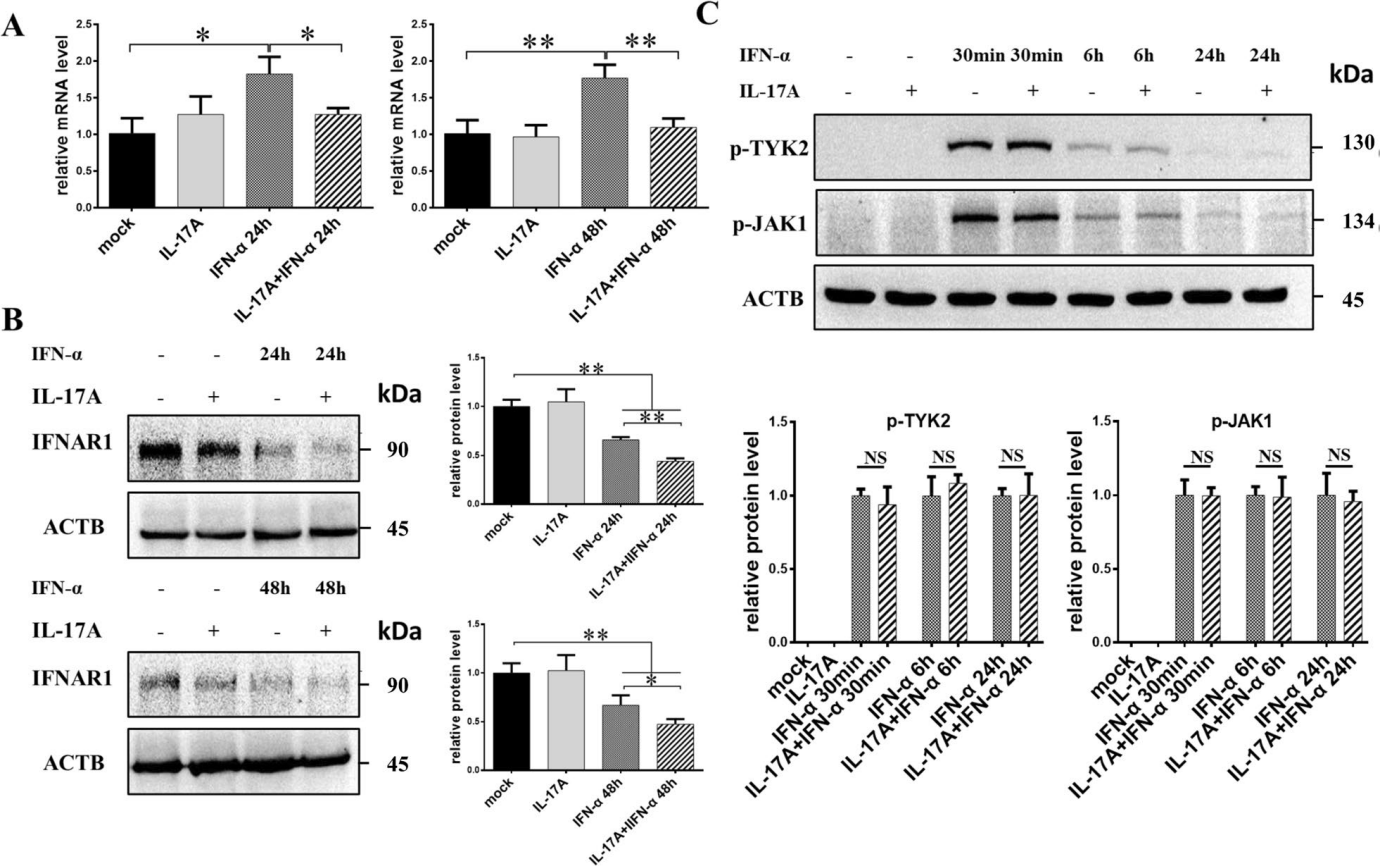
Changes in expression of IFN-α receptor 1 (IFNAR1) and phosphorylation of TYK2 and JAK1 after IL-17A pretreatment. **A**, **B** HepG2-HBV1.3 cells were pretreated with or without 50 ng/ml IL-17A for 24 h, followed by co-treatment with or without 1000 IU/ml IFN-α for 24 or 48 h. Total cellular RNA was extracted for detection of IFNAR1 mRNA by RT-qPCR (**A**), and cell lysates were prepared for assay of IFNAR1 protein by western blotting (**B**). Relative expression of mRNA or protein was shown as fold-change compared to the mock-treated cells. **C** HepG2-HBV1.3 cells were pretreated with or without 50 ng/ml IL-17A for 24 h, followed by co-treatment with or without 1000 IU/ml IFN-α for 30 min, 6 h or 24 h. Whole cell lysates were collected for assay of phosphorylation of TYK2 and JAK1 by western blotting. Relative expression in group of IL-17A pretreatment was shown as fold-change compared to its corresponding IFN-α group at each timepoint. All data were shown as mean ± standard deviation (SD) (error bars) from at least 3 independent experiments. p < 0.05 is considered statistically significant. *p < 0.05, **p < 0.01 between two indicated groups

The above results implied that IL-17A pretreatment attenuated the anti-HBV activity of IFN-α. Next, we explored the mechanism by which IL-17A affects IFN-α signaling. As IFN-α receptor 1 (IFNAR1) is the first compartment of the IFN-α signaling pathway, changes in its expression were first observed. As shown in Fig. 3A and B, treatment with IFN-α alone for 24 h or 48 h significantly increased the mRNA expression of IFNAR1 but decreased the IFNAR1 protein level. IL-17A treatment alone did not significantly affect the mRNA or protein expression level of IFNAR1. Moreover, compared to those cells upon IFN-α treatment alone, the mRNA and protein levels of IFNAR1 were significantly reduced in HepG2-HBV1.3 cells with 50 ng/ml IL-17A pretreatment for 24 h followed by cotreatment with IFN-α for 24 h or 48 h.

TYK2 and JAK1 are associated with the IFN-α receptor, and their phosphorylation mediates signal transfer from the IFN-α receptor to the cytoplasm. Phosphorylation of TYK2 and JAK1 was investigated at different time points after IFN-α stimulation with or without IL-17A pretreatment for 24 h. However, our data showed that IL-17A pretreatment had no significant effect on p-TYK2 or p-JAK1 levels at 30 min, 6 h, or 24 h after IFN-α stimulation (Fig. 3C).

### IL-17A pretreatment inhibited IFN-α-induced STAT1/2 phosphorylation and IRF9 expression

**Fig. 4:**
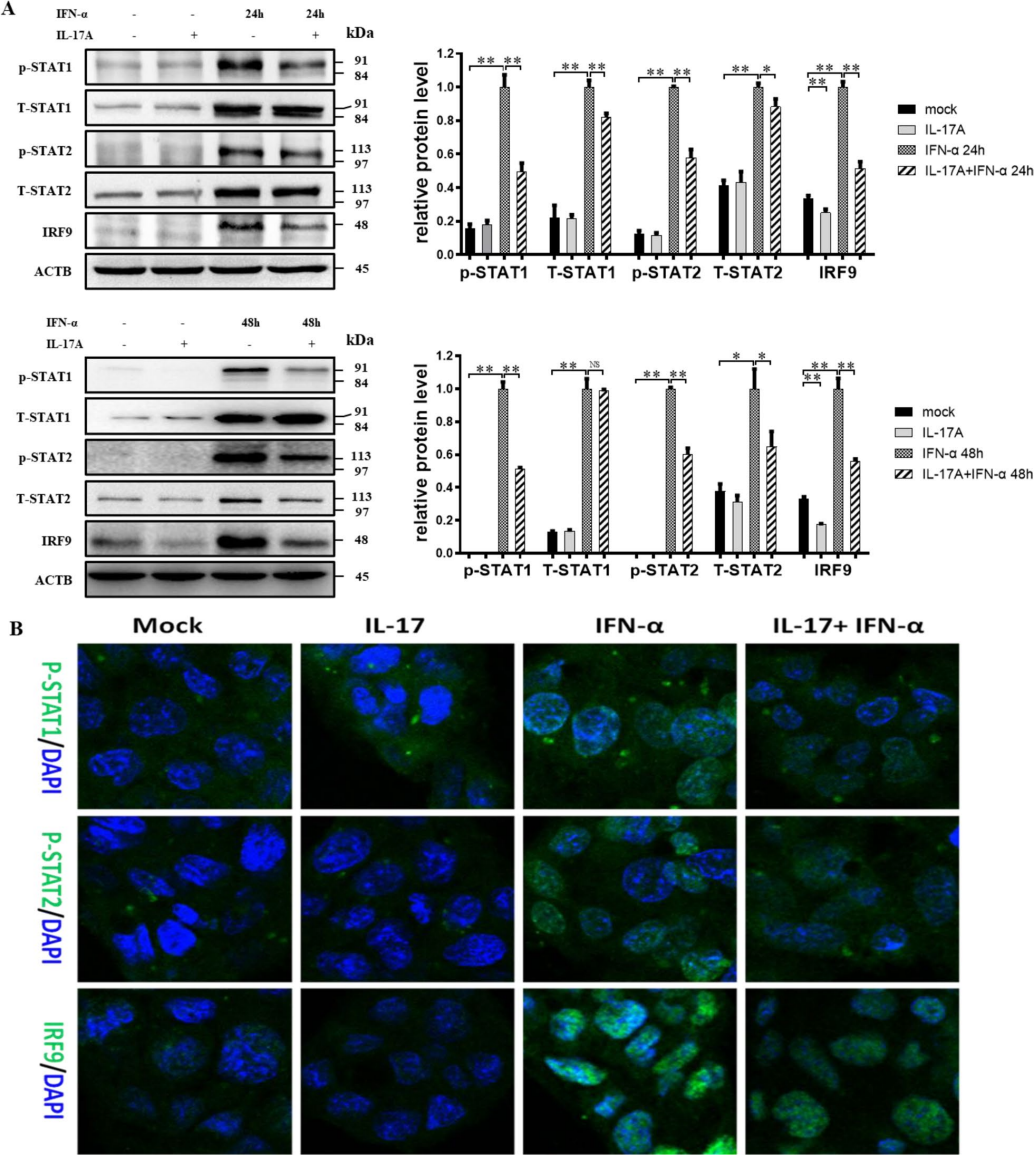
IL-17A pretreatment inhibited IFN-α-induced STAT1/2 phosphorylation and IRF9 expression. **A** HepG2-HBV1.3 cells were pretreated with or without 50 ng/ml IL-17A for 24 h, followed by co-treatment with or without 1000 IU/ml IFN-α for 24 or 48 h. Cell lysates were collected for assay of p-STAT1, T-STAT1, p-STAT2, T-STAT2 and IRF9 protein by western blotting. Relative expression was shown as fold-change compared to group of IFN-α treatment alone. Data were shown as mean ± standard deviation (SD) (error bars) from at least 3 independent experiments. p < 0.05 is considered statistically significant. *p < 0.05, **p < 0.01 between two indicated groups. **B** HepG2-HBV1.3 cells were pretreated with or without 50 ng/ml IL-17A for 24 h, followed by co-treatment with or without 1000 IU/ml IFN-α for 48 h. Cell slides were processed for immunofluorescent staning and fluorescence microscopy imaging. The merge images displayed positive expression of p-STAT1, p-STAT2 and IRF9 (green) in cell nucleus (in blue by DAPI staining)

The critical step of IFN-α signaling is forming the ISGF3 transcriptional complex (p-STAT1/p-STAT2/IRF9), which can translocate to the nucleus and induce the transcription of target genes. Here, changes in the expression and subcellular localization of p-STAT1, p-STAT2, and IRF9 were analyzed by WB and laser scanning confocal microscopy. The WB results showed that compared to that in the mock-treated HepG2-HBV1.3 cell group, IL-17A treatment alone did not significantly change the expression of total (T)-STAT1, p-STAT1, T-STAT2, or p-STAT2, but significantly decreased IRF-9 expression. IFN-α treatment alone for 24 h and 48 h induced strong expression of T-STAT1, p-STAT1, T-STAT2, p-STAT2, and IRF9, but these inductions were significantly reduced in the presence of IL-17A pretreatment for 24 h (except for T-STAT1) (Fig. 4A). In addition, laser scanning confocal microscopy images further verified the inhibitory effects of IL-17A pretreatment on the expression of p-STAT1, p-STAT2, and IRF9. The merged fluorescence images displayed strong positive p-STAT1, p-STAT2, and IRF9 expression in the nuclei of HepG2-HBV1.3 cells stimulated with IFN-α alone. In contrast, remarkably decreased expression of p-STAT1, p-STAT2 and IRF9 was observed in cells of the IL-17A-pretreatment group (Fig. 4B).

### IL-17A pretreatment suppressed IFN-α-stimulated antiviral ISGs expression but increased regulatory ISGs expression

**Fig. 5:**
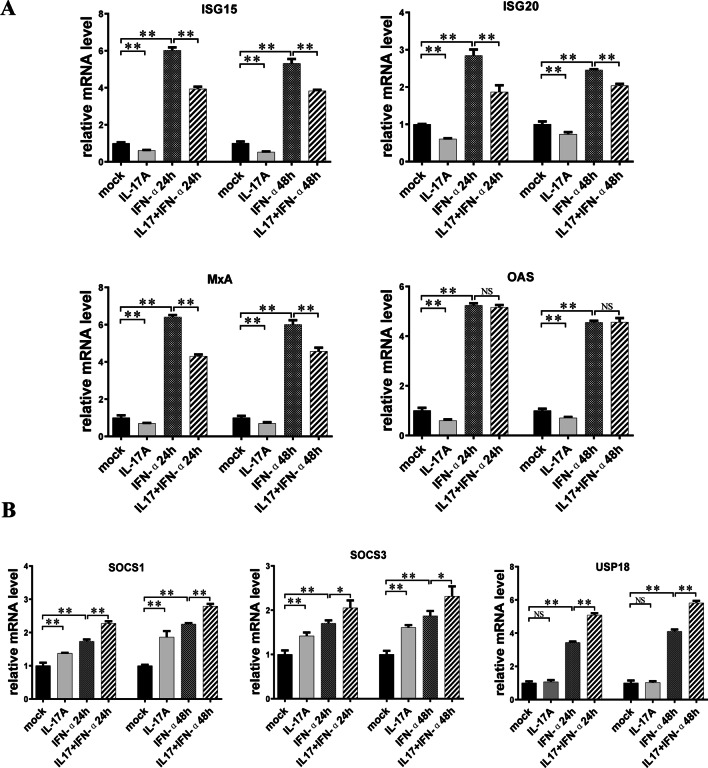
Effect of IL-17A pretreatment on the expression of anti-HBV or regulatory IFN-stimulated genes (ISGs). HepG2-HBV1.3 cells were pretreated with or without 50 ng/ml IL-17A for 24 h, followed by co-treatment with or without 1000 IU/ml IFN-α for 24 or 48 h. Total cellular RNA were extracted for detection of mRNA expression of anti-HBV ISGs (ISG15, ISG20, MxA and OAS) (**A**) and regulatory ISGs (SOCS1, SOCS3 and USP18) (**B**) by RT-qPCR. Relative expression of mRNA was shown as fold-change compared to the mock-treated cells. Data were shown as mean ± standard deviation (SD) (error bars) from at least 3 independent experiments. p < 0.05 is considered statistically significant. *p < 0.05, **p < 0.01 between two indicated groups

The activated ISGF3 transcriptional complex is transferred into the nucleus, binds ISRE in the promoters of ISGs, and stimulates their transcription. Some ISGs have been identified to have antiviral functions. Therefore, we further assessed mRNA levels of the antiviral ISGs: ISG15, ISG20, MxA, and OAS, which were reported to be positively related to the anti-HBV effect of IFN-α. As shown in Fig. 5A, the mRNA expression of ISG15, ISG20, MxA, and OAS was significantly upregulated by IFN-α stimulation but downregulated remarkably by treatment with IL-17A alone. Moreover, IL-17A pretreatment significantly decreased the IFN-α-induced expression of these antiviral ISGs, except OAS.

In addition to these antiviral ISGs, we detected the expression of other ISGs that served as negative regulators of IFN-α signaling: SOCS1, SOCS3, and USP18. As shown in Fig. 5B, the mRNA expression of SOCS1 and SOCS3 was significantly upregulated in the presence of either IL-17A or IFN-α alone. IL-17A pretreatment followed by IFN-α stimulation further increased SOCS1 and SOCS3 expression compared to IFN-α stimulation alone. Although IL-17A treatment alone didn’t significantly change the expression of USP18, IL-17A pretreatment could further enhance IFN-α-induced expression of USP18.

We further observed changes in the inhibitory effects of IL-17A after suppressing expression of SOCS1 and SOCS3 by small interfering RNA (siRNA). Our results showed that down-regulation of SOCS1 or SOCS3 expression decreased the inhibitory effects of IL-17A, and restored the anti-HBV activity of IFN-α (Additional file 6: Fig. S4).

### Suppression of the IL-17A pathway by a TRAF6 inhibitor reversed the inhibitory effect of IL-17A on IFN-α-induced ISGF3 activation and the anti-HBV activity of IFN-α

**Fig. 6:**
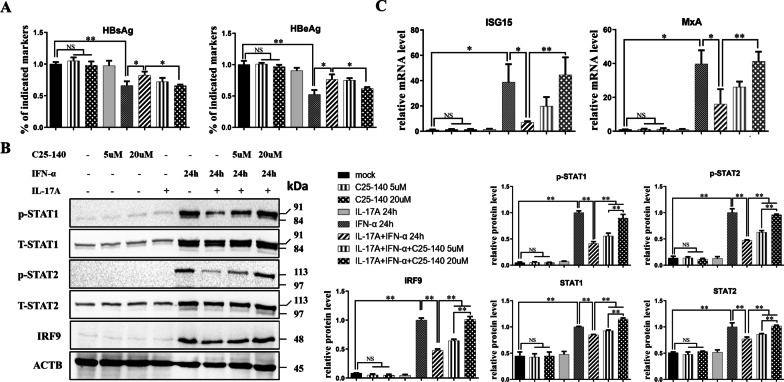
Repression of IL-17A pathway by TRAF6 inhibitor reversed the inhibitory effect of IL-17A on IFN-α-induced ISGF3 activation and anti-HBV activity. HepG2-HBV1.3 cells were pre-incubated with or without 5 uM or 20 uM TRAF6 inhibitor C25-140 for 6 h, then cells were cultured for another 24 h in this medium with or without supplementary 50 ng/ml IL-17A, and followed by co-treatment with or without 1000 IU/ml IFN-α for 24 h. **A** Culture supernatants were collected for assay of levels of HBsAg and HBeAg by ELISA. Data were displayed as a percentage of the values obtained for mock-treated cells. **B** Cell lysates were prepared for detection of protein expression by western blotting. Relative expression of protein was shown as fold-change in comparison with the cells treated with IFN-α alone. **C** Total cellular RNA was extracted for detection of mRNA expression of anti-HBV ISGs (ISG15 and MxA) by RT-qPCR. Relative expression of mRNA was shown as fold-change compared to the mock-treated cells. All data were shown as mean ± standard deviation (SD) (error bars) from at least 3 independent experiments. p < 0.05 is considered statistically significant. *p < 0.05, **p < 0.01 between two indicated groups

TRAF6 was identified as a key signal adaptor in the IL-17A pathway and was associated with the pro-inflammatory function of IL-17A [21]. To further verify the inhibitory effect of IL-17A on IFN-α signaling, we used the TRAF6 inhibitor C25-140 to suppress IL-17A pro-inflammatory signaling and evaluated changes in the expression of HBV markers, ISGF3, and antiviral ISGs. The mock group was set as the negative control (or vehicle control), which was treated with 0.1% DMSO only. As shown in Fig. 6A, the culture supernatant levels of HBsAg and HBeAg in the group of 20 µM C25-140 preincubation (IL-17A + IFN-α + C25-140 20 µM group) were significantly lower than the group of IL-17A pretreatment (IL-17A + IFN-α group). Consistent with the results above, IL-17A pretreatment significantly decreased the expression of p-STAT1, p-STAT2, and IRF9 compared to that in the group treated with IFN-α alone. And preincubation with C25-140 at both 5 µM and 20 µM restored the suppression of p-STAT1, p-STAT2, or IRF9 expression by IL-17A pretreatment (Fig. 6B). Furthermore, these restorations by C25-140 preincubation were more remarkable at high concentration (20 µM) than low concentration (5 µM), implying that these effects of C25-140 were dose-dependent. Accordingly, the inhibition of IFN-α-induced expression of anti-HBV ISGs (ISG15 and MxA) by IL-17A pretreatment was significantly restored by 20 µM C25-140 preincubation (Fig. 6C). Moreover, our results also showed that incubation with 5 µM or 20 µM C25-140 alone did not significantly affect the expression of HBV markers, ISGF3, or antiviral ISGs.

## Discussion

IL-17A is a pro-inflammatory cytokine mainly produced by Th17, Tc17 or γδ T cells. IL-17A has been shown to play critical roles in various processes, including host defense against pathogens [22–25], inflammatory and autoimmune diseases [26, 27] and tumor development [28, 29].Although the role of IL-17A has been sufficiently explored in many viral infectious diseases, its function in viral hepatitis has not been fully clarified. Some studies demonstrated that elevated level of IL-17A was associated with virus clearance and disease resolution in hepatitis induced by adenovirus or hepatitis C virus (HCV) [30–32]. However, a study for chronic HBV infection showed that the level of IL-17A was positively correlated with HBsAg and HBeAg expression [33]. And high IL-17A expression in chronic HBV infection contributed to the development of liver fibrosis and cirrhosis [34, 35]. Furthermore, an elevated level of IL-17A was also found to be involved in the immunosuppressive environment in chronic HBV infection, which was conducive to the persistence of HBV [36]. IFN-α has been broadly used against viral infection, but chronic hepatitis B (CHB) patients have an inadequate response to IFN-α treatment. However, the relationship between elevated levels of IL-17A and the anti-HBV activity of IFN-α is unconfirmed yet.

Analysis of the GEO database is a useful way to obtain valuable information for our research. Although we found only one dataset (GEO89610), containing data from human liver hepatocellular carcinoma cells treated with IL-17A, the results of gene set enrichment analysis of DEGs strongly suggested that IL-17A negatively regulates the IFN-α pathway.

In our study, IL-17A treatment alone for 24 h didn’t show any significant effect on HBV expression in HepG2-HBV1.3 cells. While in the study of Wang et al. in HepG2.2.15 cells, a significant inhibitory effect of IL-17A (1 ng/ml) on HBV replication was observed under a longer duration time (120 h) [37]. We speculate that these differences were due to a different duration time.

Moreover, our results firstly exhibited that high IL-17A concentrations (25, 50, 100 ng/ml) significantly attenuated the anti-HBV effect of IFN-α. Interestingly, slightly lower levels (but not significant) of supernatant HBeAg were found in HepG2-HBV1.3 cells co-treated with 0.2 or 1 ng/ml IL-17A and IFN-α when compared to IFN-α treatment alone. We have reviewed lots of clinical studies about IL-17A expression in peripheral blood and liver of patients with chronic hepatitis B (CHB). Most of these studies found that serum IL-17A levels in CHB patients were significantly higher than healthy controls, but the values varied a lot among these studies (from 5.21 to 190.1 pg/ml) [3]. Some other studies also showed that intrahepatic expression of IL-17A or IL-17A-related chemokines was much higher in CHB patients than that in the controls [9, 10]. Furthermore, in a recent study by Ribeiro, et al., chronic hepatitis B displayed a significantly higher level of serum IL-17A than acute hepatitis B [38]. Moreover, an increase of IL-17A was demonstrated to be related to liver cirrhosis and hepatocarcinoma [39], and high IL-17A in local tissues could induce chronic inflammatory and immunosuppressive environment [40]. Therefore, slightly elevated IL-17A might exert a synergistic anti-viral effect during acute hepatitis B, while sustained high levels of IL-17A in chronic hepatitis B might involve in HBV persistence and disease progression. On the other hand, a significant suppressive effect was found after IL-17A pretreatment for 12 h and 24 h. However, when the IL-17A pretreatment time was extended to 48 h, the effects of IL-17A were not significant anymore. From our results that IL-17A had no significant effects on cell viability of HepG2-HBV1.3 cells, we speculated that this might be caused by cell hyporesponsiveness after prolonged exposure to IL-17A.

We further explored the underlying mechanism by which IL-17A regulates IFN-α signaling by observing changes in the critical signaling cascade components. Our results showed that the mRNA and protein expression of IFNAR1 decreased more sharply in the IL-17A-pretreatment group than in the group treated with IFN-α alone, but the expression of phosphorylated JAK1 and TYK2 did not change significantly. As one of the regulatory mechanisms, IFNAR1 undergoes phosphorylation and ubiquitination of its degron region, followed by lysosomal degradation after IFN-α stimulation [41, 42]. Several phosphokinases, such as casein kinase 1 alpha 1 (CK1α), and protein kinase D2 (PKD2), are associated with this process [43–45]. We also detected the expression of CK1α and PKD2 and found it was increased in the IL-17A-pretreatment group (data not shown). This indicated that IL-17A pretreatment exacerbated the IFN-α-induced ligand-dependent degradation of IFNAR1. Moreover, we noticed that IL-17A pretreatment did not significantly change the levels of phosphorylated JAK1 or TYK2, which was probably due to the effective phosphorylation of JAK1 or TYK2 by even a small number of receptors, or transient and rapid phosphorylation of JAK1 or TYK2 immediately after IFNAR crosslinking. We further used JAK1 inhibitor to investigate IL-17A effects on IFN-α signaling, and found that the inhibition of JAK1 further increased the suppression effects of IL-17A (Additional file 7: Fig. S5).

In the canonical IFN-α signaling pathway, the activation of JAK1 and TYK2 phosphorylates STAT1 and STAT2, leading to their dimerization, after which p-STAT1 and p-STAT2 form the ISGF3 transcriptional complex by binding IRF9. Our results showed that IL-17A pretreatment significantly decreased the expression of p-STAT1, p-STAT2, and IRF9 proteins. It is well known that ISGF3 can translocate to the nucleus and bind IFN stimulated response elements (ISREs), and then induce hundreds of ISGs. Some ISGs are reported to be related to the anti-HBV activity of IFN-α, such as ISG15, ISG20, OAS1, or MX1 [46–51]. Our results also showed that IL-17A pretreatment significantly decreased the expression of these antiviral ISGs.

Some other ISGs, such as SOCS1, SOCS3, or USP18, are identified to be involved in the negative feedback loop of IFN signaling to avoid cell overstimulation by IFN-α [52–55]. Interestingly, these regulatory ISGs (especially SOCS1 or SOCS3) can be induced not only by IFN-α but also by other inflammatory cytokines [56]. In our study, IL-17A alone upregulated the mRNA expression of SOCS1 and SOCS3, and IL-17A pretreatment markedly upregulated IFN-α-induced levels of SOCS1, SOCS3, and USP18. Wang et al. also showed that IL-17A could upregulate the expression of SOCS3 in natural killer cells [57]. Furthermore, IL-6 and TNF-α are target genes of IL-17A [58], which promotes the expression of SOCS3 by activating STAT3 in hepatocytes [59] and enhances USP18 expression in Huh7.5 cells and primary murine hepatocytes undergone inflammatory stimuli (TNF-α and lipopolysaccharide (LPS)) [60], respectively. And our results further showed that suppression of SOCS1 and SOCS3 expression decreased the inhibitory effects of IL-17A on IFN-α-induced anti-HBV activity (Additional file 6: Fig. S4). Therefore, IL-17A pretreatment might increase the expression of the SOCS gene and subsequently reduce cell response to IFN-α by inhibition of STAT1/STAT2/IRF9 and target genes. However, the mechanism by which IL-17A upregulates SOCS expression remains unclear, and further research is needed.

TRAF6 was identified as a critical signal adaptor in the IL-17A pathway. Our results showed that suppressing the IL-17A pathway by a TRAF6 inhibitor reversed the inhibitory effect of IL-17A on the anti-HBV activity of IFN-α. That is, attenuated IFN-α signaling mediated by IL-17A pretreatment is at least partially mediated by activation of TRAF6 signaling.

## Conclusion

**Fig. 7:**
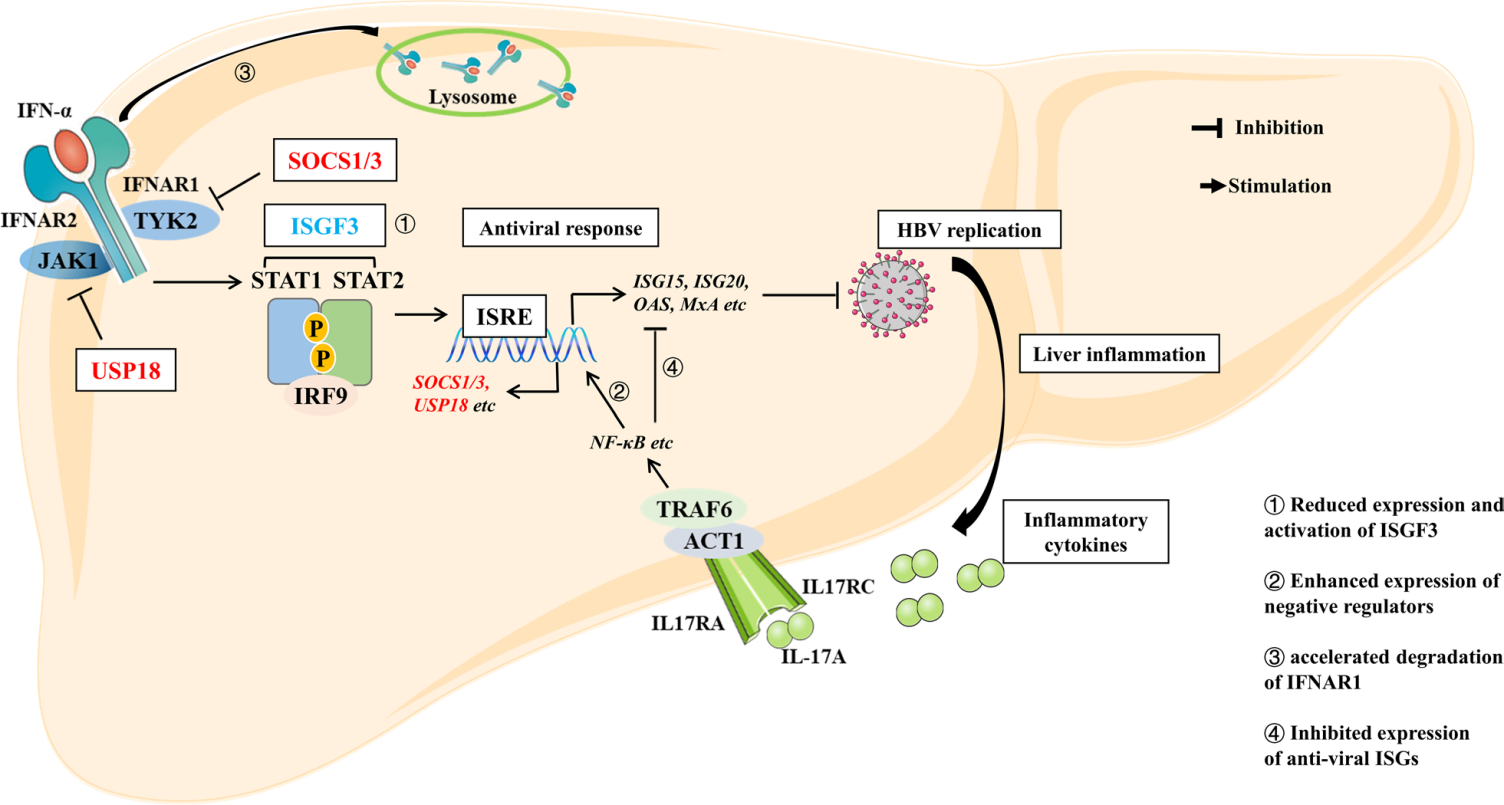
A model of regulatory effects of IL-17A on anti-HBV activity of IFN-α. IFN-α exerts anti-HBV activity by activating JAK-STAT signaling pathway including STAT1 and STAT2 phosphorylation, transcription factor ISGF3 formation, and at last antiviral ISGs expression. Various pro-inflammatory cytokines cross-regulate the IFN-α response by altering the expression and activation of IFN-α signaling components. The pro-inflammatory function of IL-17A is primarily mediated by NF-κB signal activated by ACT1-TRAF6. In our study, IL-17A attenuates IFN-α-mediated anti-HBV activity through reduced expression and activation of ISGF3. Furthermore, IL-17A also enhances expression of negative regulators, including SOCS1/3 and USP18, inhibits expression of anti-viral ISGs and accelerates degradation of IFNAR1

In conclusion, our data provide novel findings suggesting that IL-17A attenuated IFN-α-mediated anti-HBV activity in HBV-expressing HepG2 cells through reducing the expression of ISGF3 and enhancing the degradation of IFNAR1, as shown in Fig. 7. However, this study included only in vitro experiments, and these in vitro observations should be verified in in vivo models and clinical studies. If the correlation between high IL-17A levels and poor response to IFN-α treatment is confirmed by large-scale clinical studies, the use of IL-17A neutralizing antibodies (eg. Cosentyx/Secukinumab, a monoclonal antibody that has been approved for the treatment of moderate to severe psoriasis) could be considered in CHB patients with both high IL-17A level and poor IFN-α response. Altogether, blockade of excessive IL-17A signaling in the liver might be a novel therapeutic strategy to improve treatment efficacy in patients with CHB.

## Supplementary Information

**Additional file 1: Table S1**. Primers sequences used in the quantitative real-time PCR analysis.

**Additional file 2: Table S2**. Representative top IL-17A-regulated genes in Huh7.5 cells from GSE89610 dataset.

**Additional file 3: Figure S1**. Levels of HBsAg and HBeAg in culture supernatants from HepG2-HBV1.3 cells at 48, 72, 96 and 120 hours after transfection with the recombinant plasmid pCDNA3.1 HBV1.3.

**Additional file 4: Figure S2**. Changes in gene expression and type-I IFN pathway in IL-17A-treated Huh7.5 cells from GSE89610 dataset.

**Additional file 5: Figure S3**. Effect of IFN-α or IL-17A treatment alone on cell viability or HBV expression of HepG2-HBV1.3 cells.

**Additional file 6: Figure S4**. Suppression of SOCS1 and SOCS3 expression by siRNA decreased the inhibitory effects of IL-17A on IFN-α-induced anti-HBV activity.

**Additional file 7: Figure S5**. Inhibitor-mediated reduced JAK1 activity abolished the effect of IL-17A on the anti-HBV activity of IFN-α.

## Data Availability

The datasets used and/or analyzed during the current study are available from the corresponding author on request.

## Abbreviations

HBV: Hepatitis B virus
HBsAg: Hepatitis B surface antigen
HBeAg: Hepatitis B e antigen
HBcAg: Hepatitis B core antigen
CHB: Chronic hepatitis B
HC: Healthy control
IFN-α: Interferon-alpha
IL: Interleukin
NAs: Nucleos(t)ide analogs
GEO: Gene expression omnibus
ISGF3: Interferon-stimulated transcription factor 3
p-STAT1: Phosphorylated signal transducer and activator of transcription 1
p-STAT2: Phosphorylated signal transducer and activator of transcription 2
IRF9: Interferon regulatory factor 9
IFNAR1: Interferon alpha and beta receptor subunit 1
p-TYK2: Phosphorylated tyrosine kinase 2
p-JAK1: Phosphorylated janus kinase 1
ISRE: IFN-stimulated response elements
ISG: Interferon stimulate genes
OAS: Oligoadenylate synthetase
Mx1: Mx dynamin-like GTPase 1
SOCS1: Suppressor of cytokine signalling 1
SOCS3: Suppressor of cytokine signalling 3
USP18: Ubiquitin specific protease 18
TRAF6: TNF receptor associated factor 6
PKD2: Protein kinase D2
CK1α: Casein kinase 1 alpha 1
PTM: Post-translational modification
