# Supplementary material for: Interleukin-17A pretreatment attenuates the anti-hepatitis B virus efficacy of interferon-alpha by reducing activation of the interferon-stimulated gene factor 3 transcriptional complex in hepatitis B virus-expressing HepG2 cells
Source: Virol J. 2022 Feb 10;19:28. doi: 10.1186/s12985-022-01753-x (PMC8830041; doi:10.1186/s12985-022-01753-x)
Supplement: Supplementary file 1 — Additional file 1: Table S1. Primers sequences used in the quantitative real-time PCR analysis. [file 12985_2022_1753_MOESM1_ESM.docx]

**Table S1.** **Primers sequences used in the quantitative real-time PCR analysis.**

| Gene | Forward primer (5’-3’) | Reverse primer (5’-3’) |
| --- | --- | --- |
| GAPDH | GGTGGTCTCCTCTGACTTCAACA | GTTGCTGTAGCCAAATTCGTTGT |
| HBsAg | GTGTCTGCGGCGTTTTATCA | GACAAACGGGCAACATACCTT |
| HBcAg | TAGCTACCTGGGTGGGTGTT | AAGCTGGAGGAGTGCGAATC |
| MX1 | AGGACCATCGGAATCTTGAC | TCAGGTGGAACACGAGGTTC |
| ISG15 | GGACAAATGCGACGAACCTCT | GCCCGCTCACTTGCTGCTT |
| ISG20 | GCGGCTACACAATCTACGACAC | CAGGCTGTTCTGGATGCTCTTG |
| OAS | AGTTCTCCACCTGCTTCACA | TAGGCGGATGAGGCTCTTG |
| SOCS1 | GGAACTGCTTTTTCGCCCTTA | AGCAGCTCGAAGAGGCAGTC |
| SOCS3 | GTCCCCCCAGAAGAGCCTATTA | TTGACGGTCTTCCGACAGAGAT |
| USP18 | ACCTGCTGCCTTAACTCCTTGA | CGCTTCTCCTCTGCTCGTCA |
| IFNAR1 | CGCCTGTGATCCAGGATTATCC | TGGTGTGTGCTCTGGCTTTCAC |
