# Supplementary material for: Interleukin-17A pretreatment attenuates the anti-hepatitis B virus efficacy of interferon-alpha by reducing activation of the interferon-stimulated gene factor 3 transcriptional complex in hepatitis B virus-expressing HepG2 cells
Source: Virol J. 2022 Feb 10;19:28. doi: 10.1186/s12985-022-01753-x (PMC8830041; doi:10.1186/s12985-022-01753-x)
Supplement: Supplementary file 2 — Additional file 2: Table S2. Representative top IL-17A-regulated genes in Huh7.5 cells from GSE89610 dataset. [file 12985_2022_1753_MOESM2_ESM.docx]

**Table S2.** **Representative top IL-17A-regulated genes in Huh7.5 cells from GSE89610 dataset.**

| **Top 50 up-regulated genes** | | | | | |
| --- | --- | --- | --- | --- | --- |
| **Gene.symbol** | **Log2FC**  **(IL-17A/MOCK)** | **p-value** | **Gene.symbol** | **Log2FC**  **(IL-17A/MOCK)** | **p-value** |
| **NTS** | **3.9476** | **0.0000** | **UTP20** | **2.4852** | **0.0000** |
| **DKK1** | **3.9168** | **0.0000** | **DTL** | **2.4663** | **0.0000** |
| **LGR5** | **3.7498** | **0.0000** | **ORM2** | **2.4443** | **0.0000** |
| **SLC6A14** | **3.6058** | **0.0000** | **RRM2** | **2.4111** | **0.0000** |
| **CLSPN** | **3.4879** | **0.0000** | **GINS4** | **2.4077** | **0.0000** |
| **NME1** | **3.1274** | **0.0000** | **XRCC2** | **2.4067** | **0.0000** |
| **RABL2A** | **2.8835** | **0.0001** | **MT1X** | **2.3984** | **0.0000** |
| **RABL2B** | **2.8835** | **0.0001** | **ANXA1** | **2.3829** | **0.0000** |
| **NCAPG** | **2.8507** | **0.0000** | **HSPH1** | **2.3727** | **0.0001** |
| **MCM10** | **2.8224** | **0.0000** | **FAM111B** | **2.3367** | **0.0000** |
| **AGMAT** | **2.8065** | **0.0000** | **RRS1** | **2.3055** | **0.0000** |
| **NRK** | **2.7845** | **0.0000** | **GINS2** | **2.2966** | **0.0000** |
| **CDC6** | **2.7671** | **0.0000** | **CENPF** | **2.2938** | **0.0000** |
| **ASPM** | **2.7024** | **0.0000** | **DDIAS** | **2.2685** | **0.0000** |
| **CDC25A** | **2.6746** | **0.0000** | **BUB1B** | **2.2435** | **0.0000** |
| **CDC45** | **2.6388** | **0.0000** | **FABP5** | **2.2392** | **0.0000** |
| **EXO1** | **2.6289** | **0.0000** | **FABP5P3** | **2.2392** | **0.0000** |
| **MCM4** | **2.6216** | **0.0000** | **PKI55** | **2.2270** | **0.0000** |
| **HIST1H2AB** | **2.6059** | **0.0000** | **GEN1** | **2.2009** | **0.0000** |
| **PUS7** | **2.5707** | **0.0000** | **HELLS** | **2.1969** | **0.0000** |
| **MT2A** | **2.5468** | **0.0000** | **ENC1** | **2.1793** | **0.0000** |
| **HIST1H1B** | **2.5280** | **0.0000** | **GINS1** | **2.1689** | **0.0000** |
| **BLM** | **2.5216** | **0.0000** | **ESCO2** | **2.1624** | **0.0000** |
| **DNA2** | **2.5023** | **0.0000** | **TYMS** | **2.1602** | **0.0000** |
| **HIST1H2BM** | **2.4935** | **0.0000** | **MRTO4** | **2.1494** | **0.0000** |

Continued:

| **Top 50 down-regulated genes** | | | | | |
| --- | --- | --- | --- | --- | --- |
| **Gene.symbol** | **Log2FC**  **(IL-17A/MOCK)** | **p-value** | **Gene.symbol** | **Log2FC**  **(IL-17A/MOCK)** | **p-value** |
| **ACSM3** | **-3.7423** | **0.0000** | **AKR1C2** | **-2.3231** | **0.0000** |
| **FOS** | **-3.6027** | **0.0000** | **DDIT3** | **-2.3215** | **0.0000** |
| **MIR21** | **-3.3182** | **0.0000** | **FAXDC2** | **-2.2974** | **0.0000** |
| **LBP** | **-3.2811** | **0.0000** | **SERPINC1** | **-2.2964** | **0.0000** |
| **ACSS2** | **-3.2254** | **0.0000** | **DUSP1** | **-2.2630** | **0.0000** |
| **FOSB** | **-3.1393** | **0.0000** | **SLC15A1** | **-2.2562** | **0.0000** |
| **SULT1C2** | **-3.0992** | **0.0000** | **PCK1** | **-2.2341** | **0.0000** |
| **SLC6A4** | **-3.0210** | **0.0000** | **ADHFE1** | **-2.2124** | **0.0000** |
| **PLA1A** | **-2.9931** | **0.0000** | **GPR37** | **-2.1991** | **0.0000** |
| **CYP1A1** | **-2.9840** | **0.0000** | **IFITM10** | **-2.1563** | **0.0000** |
| **GADD45B** | **-2.8265** | **0.0000** | **GSDMB** | **-2.1498** | **0.0000** |
| **PPARGC1A** | **-2.7908** | **0.0000** | **PDE1A** | **-2.1496** | **0.0000** |
| **TCP11L2** | **-2.7429** | **0.0000** | **CREBRF** | **-2.1451** | **0.0000** |
| **DIO1** | **-2.7002** | **0.0000** | **JUN** | **-2.1349** | **0.0000** |
| **C10orf10** | **-2.6403** | **0.0000** | **FOXO4** | **-2.1110** | **0.0000** |
| **PIK3IP1** | **-2.6305** | **0.0000** | **CDH1** | **-2.1055** | **0.0000** |
| **TXNIP** | **-2.5734** | **0.0000** | **TNFSF10** | **-2.1010** | **0.0000** |
| **HSD17B14** | **-2.5511** | **0.0000** | **ACSM2B** | **-2.0954** | **0.0000** |
| **CPEB4** | **-2.5421** | **0.0000** | **EPB41L4A-AS1** | **-2.0838** | **0.0000** |
| **EGR1** | **-2.5375** | **0.0000** | **BTG2** | **-2.0532** | **0.0000** |
| **KLHL24** | **-2.4851** | **0.0000** | **TMEM150B** | **-2.0432** | **0.0000** |
| **SLC25A27** | **-2.4153** | **0.0000** | **SAT2** | **-2.0387** | **0.0000** |
| **CYP4F3** | **-2.4006** | **0.0000** | **ERP27** | **-2.0238** | **0.0000** |
| **FGL1** | **-2.4000** | **0.0000** | **PCSK9** | **-2.0049** | **0.0000** |
| **C8B** | **-2.3885** | **0.0000** | **HMGCS2** | **-2.0044** | **0.0000** |
