# Supplementary material for: Interleukin-17A pretreatment attenuates the anti-hepatitis B virus efficacy of interferon-alpha by reducing activation of the interferon-stimulated gene factor 3 transcriptional complex in hepatitis B virus-expressing HepG2 cells
Source: Virol J. 2022 Feb 10;19:28. doi: 10.1186/s12985-022-01753-x (PMC8830041; doi:10.1186/s12985-022-01753-x)
Supplement: Supplementary file 3 — Additional file 3: Figure S1. Levels of HBsAg and HBeAg in culture supernatants from HepG2-HBV1.3 cells at 48, 72, 96 and 120 hours after transfection with the recombinant plasmid pCDNA3.1 HBV1.3. [file 12985_2022_1753_MOESM3_ESM.docx]

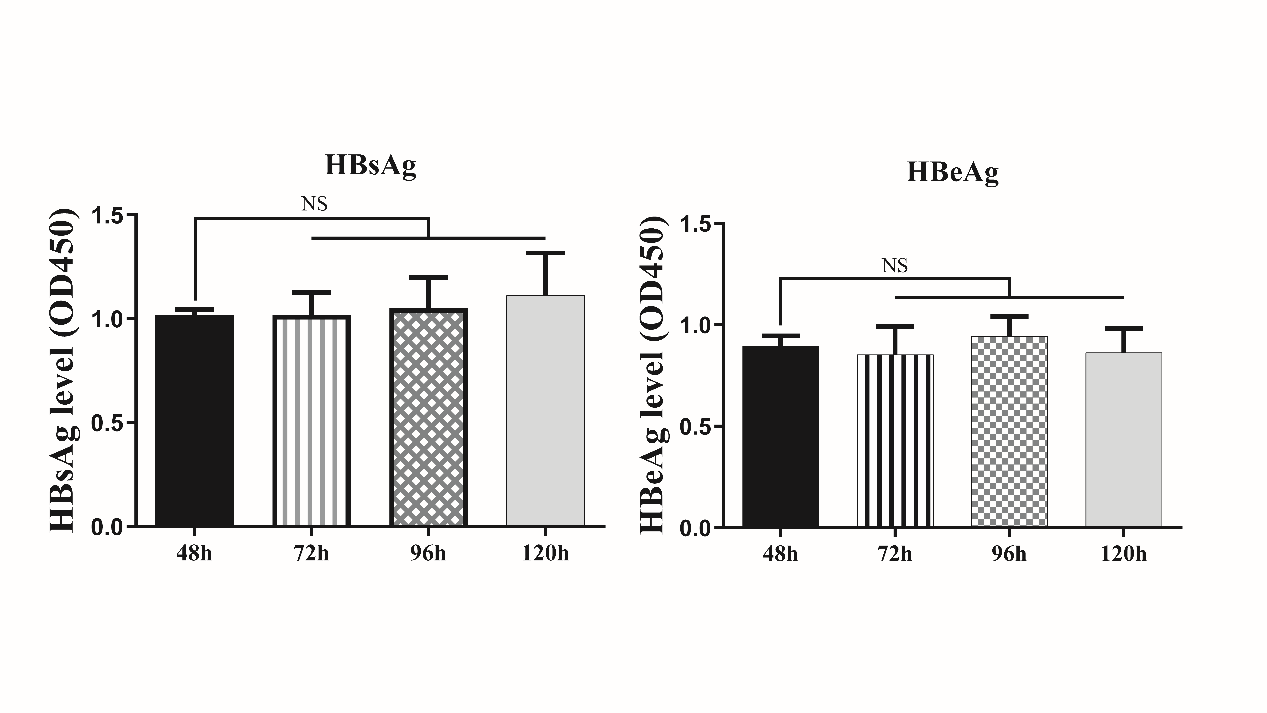


**Fig. S1.** **Levels of HBsAg and HBeAg in culture supernatants from HepG2-HBV1.3 cells at 48, 72, 96 and 120 hours after transfection with the recombinant plasmid pCDNA3.1-HBV1.3.**

Our results showed that the expression of HBsAg and HBeAg in HepG2-HBV1.3 cells has no significant change within at least 120 hours after transfection with the recombinant plasmid pCDNA3.1-HBV1.3.
