# Supplementary material for: Interleukin-17A pretreatment attenuates the anti-hepatitis B virus efficacy of interferon-alpha by reducing activation of the interferon-stimulated gene factor 3 transcriptional complex in hepatitis B virus-expressing HepG2 cells
Source: Virol J. 2022 Feb 10;19:28. doi: 10.1186/s12985-022-01753-x (PMC8830041; doi:10.1186/s12985-022-01753-x)
Supplement: Supplementary file 4 — Additional file 4: Figure S2. Changes in gene expression and type-I IFN pathway in IL-17A-treated Huh7.5 cells from GSE89610 dataset. [file 12985_2022_1753_MOESM4_ESM.docx]

**
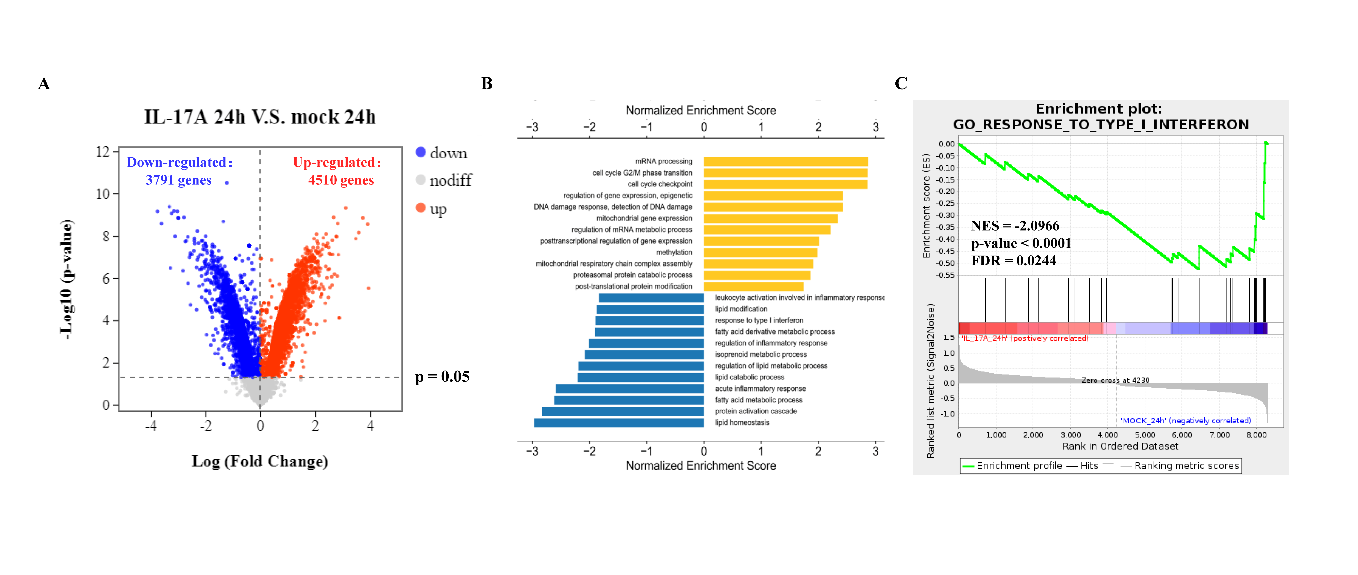
**

**Fig. S2. Changes in gene expression and type-I IFN pathway in IL-17A-treated Huh7.5 cells from GSE89610 dataset**.

GSE89610 dataset was used for the analysis of differential expressed genes (DEGs) between IL-17A-treated (24 hours) or mock-treated Huh7.5 cells. Raw data were normalized and log2 transformed with the GEO2R platform, and differences in relative gene expression between the treatment and mock groups were statistically analyzed. A: The volcano map of 8301 differentially expressed genes (DEGs) that screened with the threshold of p<0.05, in which 3791 genes are down-regulated while 4510 genes are up-regulated. To understand the most detailed biological changes, these DEGs were further analyzed by Gene Set Enrichment Analysis (GSEA) (www.omicshare.com/tools). B: Some top significantly enriched GO terms in the results of GSEA analysis; C: Results of GSEA analysis for these DEGs related to pathway of "response to type I interferon".

Our results showed that the pathway of “response to type I IFN” was significantly downregulated by IL-17A treatment (the normalized enrichment score (NES) was -2.0996, p<0.05, FDR<25%).
