# Supplementary material for: Interleukin-17A pretreatment attenuates the anti-hepatitis B virus efficacy of interferon-alpha by reducing activation of the interferon-stimulated gene factor 3 transcriptional complex in hepatitis B virus-expressing HepG2 cells
Source: Virol J. 2022 Feb 10;19:28. doi: 10.1186/s12985-022-01753-x (PMC8830041; doi:10.1186/s12985-022-01753-x)
Supplement: Supplementary file 5 — Additional file 5: Figure S3. Effect of IFN-α or IL-17A treatment alone on cell viability or HBV expression of HepG2-HBV1.3 cells. [file 12985_2022_1753_MOESM5_ESM.docx]

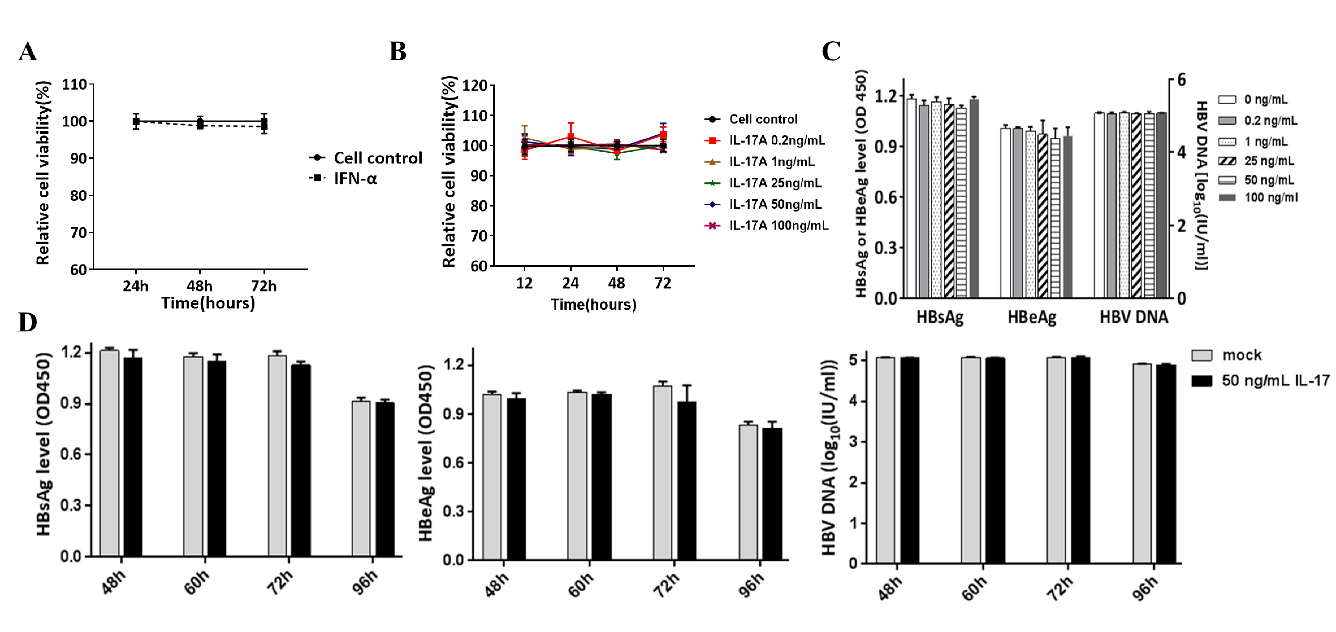


**Fig. S3.** **Effect of IFN-α or IL-17A treatment alone on cell viability or HBV expression of HepG2-HBV1.3 cells.**

A. Cell viability of HepG2-HBV1.3 cells treated with IFN-α at 1000 IU/ml for 24h, 48h or 72h was detected by CCK8 assay. B. Cell viability of HepG2-HBV1.3 cells treated with IL-17A at 0, 0.2, 1, 25, 50 or 100 ng/ml for 12h, 24h, 48h or 72h was detected by CCK8 assay. C. The levels of HBsAg, HBeAg or HBV DNA of cell culture supernatant from HepG2-HBV1.3 cells treated with IL-17A at 0, 0.2, 1, 25, 50 or 100 ng/ml for 24h. D. The levels of HBsAg, HBeAg or HBV DNA of cell culture supernatant from HepG2-HBV1.3 cells treated with 50 ng/ml IL-17A for 48h, 60h, 72h or 96h. Data were displayed as a percentage relative to mock control group. p<0.05 is considered statistically significant.

Our results showed that IL-17A or IFN-α treatment alone did not significantly affect the cell viability of HepG2-HBV1.3 cells. And the supernatant levels of HBsAg, HBeAg, or HBV DNA were not significantly changed by IL-17A alone at different concentration for different durations.
