# Supplementary material for: Interleukin-17A pretreatment attenuates the anti-hepatitis B virus efficacy of interferon-alpha by reducing activation of the interferon-stimulated gene factor 3 transcriptional complex in hepatitis B virus-expressing HepG2 cells
Source: Virol J. 2022 Feb 10;19:28. doi: 10.1186/s12985-022-01753-x (PMC8830041; doi:10.1186/s12985-022-01753-x)
Supplement: Supplementary file 6 — Additional file 6: Figure S4. Suppression of SOCS1 and SOCS3 expression by siRNA decreased the inhibitory effects of IL-17A on IFN-α-induced anti-HBV activity. [file 12985_2022_1753_MOESM6_ESM.docx]

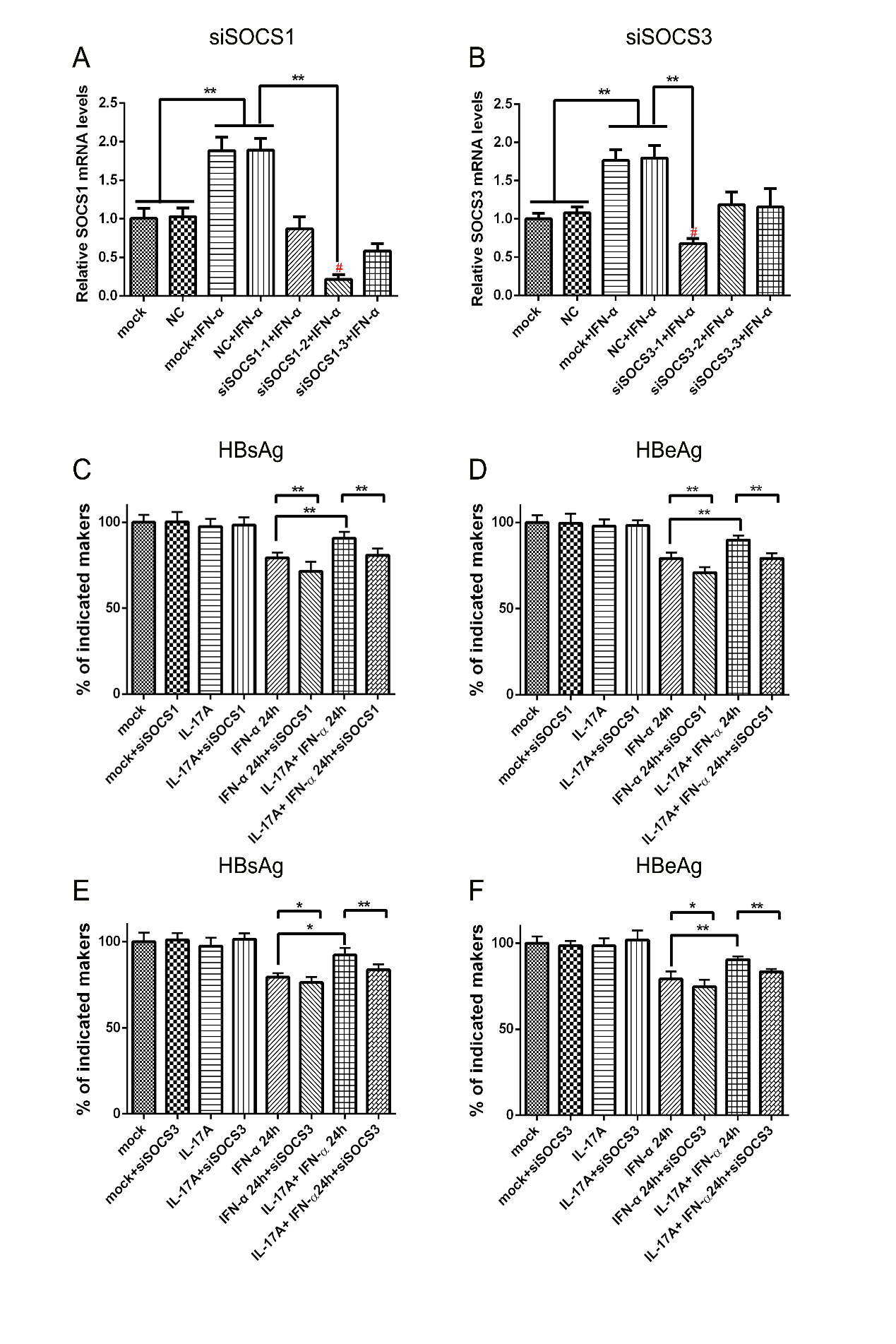


**Fig. S4. Suppression of SOCS1 and SOCS3 expression by siRNA decreased the inhibitory effects of IL-17A on IFN-α-induced anti-HBV activity.**

2.5×10^5^ HepG2-HBV1.3 cells per well of 12-well plates were plated and transfected with 30 pmol siRNA (GenePharma, China) targeting to SOCS1 (Forward: 5’-GGUUGUUGUAGCAGCUUAATT-3’, Reverse: 5’-UUAAGCUGCUACAACAA CCTT-3’), SOCS3 (Forward: 5’-UCUUCACGCUCAGCGUCAATT-3’, Reverse: 5’-UUGACGCUGAGCGUGAAGATT-3’), or negative control (NC) siRNA for 24 h using Lipofectamine 3000 transfection reagent (Invitrogen, USA) according to the protocol supplied. After that, HepG2-HBV1.3 cells were mock treated, treated with IL-17A or IFN-α alone for 24 h, or treated with IL-17A for 24 h followed by IFN-α 24 h. A. B. comparison of the inhibitory effect among three candidate siRNA sequences targeting to SOCS1 or SOCS3. Total cellular RNA was extracted for detection of SOCS1 and SOCS3 mRNA by qPCR. Relative expression of mRNA was shown as fold-change compared to the mock-treated cells. The results showed that siSOCS1-2 and siSOCS3-1 showed the best interference efficiency. So we used siSOCS1-2 and siSOCS3-1 in our later experiments. The red # symbol indicates the selected siRNA. C~F. Culture supernatants were collected for assay of levels of HBsAg and HBeAg. Data were displayed as a percentage of the values obtained for mock-treated cells. All data were shown as mean ± standard deviation (SD) (error bars) from at least 3 independent experiments. p<0.05 is considered statistically significant. * p<0.05, ** p<0.01 between two indicated groups.

Our results showed that SOCS1- and SOCS3-targeted siRNA greatly reduced the mRNA levels of SOCS1 and SOCS3, respectively. IL-17A pre-treatment significantly increased the levels of HBsAg and HBeAg compared to that in the group treated with IFN-α alone. Moreover, suppression of SOCS1 and SOCS3 expression by siRNA significantly decreased the levels of HBsAg and HBeAg, both in group treated with IFN-α alone and in IL-17A pre-treatment group.
