# Supplementary material for: Interleukin-17A pretreatment attenuates the anti-hepatitis B virus efficacy of interferon-alpha by reducing activation of the interferon-stimulated gene factor 3 transcriptional complex in hepatitis B virus-expressing HepG2 cells
Source: Virol J. 2022 Feb 10;19:28. doi: 10.1186/s12985-022-01753-x (PMC8830041; doi:10.1186/s12985-022-01753-x)
Supplement: Supplementary file 7 — Additional file 7: Figure S5. Inhibitor-mediated reduced JAK1 activity abolished the effect of IL-17A on the anti-HBV activity of IFN-α. [file 12985_2022_1753_MOESM7_ESM.docx]

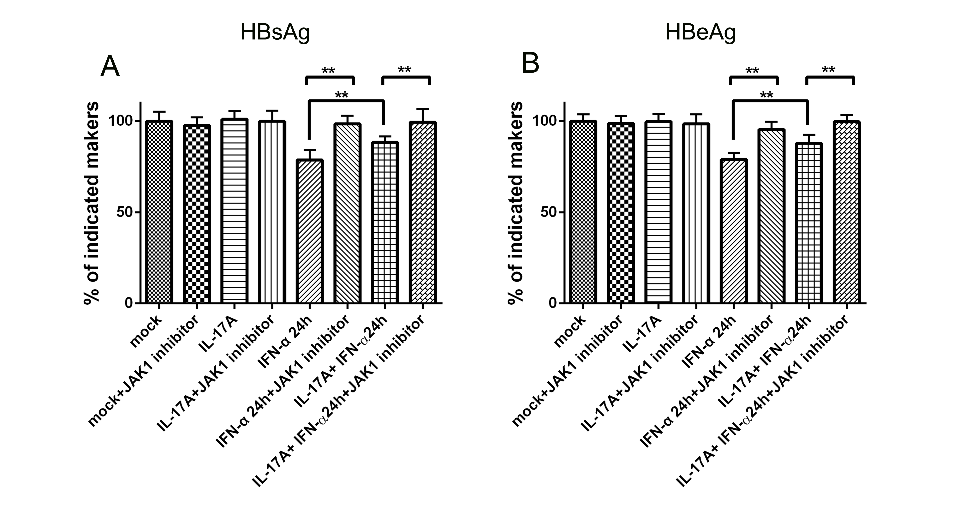


**Fig. S5.** **Inhibitor-mediated reduced JAK1 activity abolished the effect of IL-17A on the anti-HBV activity of IFN-α.**

2.5×10^5^ HepG2-HBV1.3 cells per well of 12-well plates were plated and they were first incubated with 0.05 uM JAK1 inhibitor Upadacitinib (MCE HY-19569) for 6 h. And then these cells were cultured in fresh medium and first pre-treated with IL-17A (PeproTech, UK) at 50 ng/ml for 24 h, followed by combined treatment with recombinant human IFN-α-2b (1000 IU/mL) (Anhui Anke Biotechnology, China) and IL-17A in fresh medium for another 24 h. Ultimately, levels of HBsAg and HBeAg in the cell culture supernatant were detected with commercial ELISA kits (Kehua, China) according to the manufacturer’s instructions. Data were displayed as a percentage of the values obtained for mock-treated cells. All data were shown as mean ± standard deviation (SD) (error bars) from at least 3 independent experiments. p<0.05 is considered statistically significant. * p<0.05, ** p<0.01 between two indicated groups.

Our results showed that the supernatant HBsAg and HBeAg levels were significantly increased in the IL-17A pre-treatment group than that in the group treated with IFN-α alone. Importantly, pre-incubation of JAK1 inhibitor remarkably increased HBsAg and HBeAg levels compared to that the group of IFN-α treated alone or the IL-17A pre-treatment group.
